# Cu_3_Sn joint based on transient liquid phase bonding of Cu@Cu_6_Sn_5_ core–shell particles

**DOI:** 10.1038/s41598-023-27870-3

**Published:** 2023-01-12

**Authors:** Jintao Wang, Jianqiang Wang, Fangcheng Duan, Hongtao Chen

**Affiliations:** 1 State Key Lab of Advanced Soldering and Joining, Harbin Institute of Technology, Harbin, 150001 China; 2 Sauvage Laboratory for Smart Materials, Harbin Institute of Technology (Shenzhen), Shenzhen, 518055 China

**Keywords:** Electronic devices, Atomistic models, Materials science

## Abstract

With the development of high-integration and high-power electronics, the lack of matching chip connecting materials that can withstand high temperatures has been a challenge. In this manuscript, a Cu@Cu_6_Sn_5_ core–shell bimetallic particles (approx. 1 μm in diameter) are successfully prepared and introduced as a new solder material for the packaging of power devices to obtain a Cu_3_Sn all-IMC solder joint. The joint consisted mainly of equiaxed Cu_3_Sn grains, and a small portion of columnar Cu_3_Sn grains. In columnar-type growth, Sn is the dominant diffusing species, which comes from the depletion of Sn in Cu_6_Sn_5_. The depleted Cu_6_Sn_5_ is transformed into columnar Cu_3_Sn. In equiaxed-type growth, Cu is the dominant diffusing species. Cu reacts with Cu_6_Sn_5_ to grow a Cu_3_Sn layer. This conclusion was confirmed by the orientation relationship. The equiaxed Cu_3_Sn grain nucleates at the Cu/Cu_3_Sn interface have an orientation relationship with the Cu substrate. Columnar Cu_3_Sn grains at the Cu_6_Sn_5_/Cu_3_Sn interface have an orientation relationship with Cu_6_Sn_5_.

## Introduction

With the development of high-integration and high-power electronics, there have been rapid advances in the fabrication of new power devices based on SiC, GaN and other wide bandgap semiconducting materials. SiC-based power devices have been found to operate up to 600 °C^1–3^, but the lack of matching chip connecting materials that can withstand high temperatures has been a challenge. Excessive reflow temperatures cause high thermal stress and may damage other temperature-sensitive devices in the system. Therefore, the solder material should preferably operate under low-temperature and short-reflow conditions, and the resulting solder joints can withstand higher service temperatures^4–6^.

The metallurgical reactions of Cu–Sn systems have been well studied for many years^7–9^. It involves the formation of two types of intermetallic compounds (IMCs): Cu_6_Sn_5_ and Cu_3_Sn. Cu_3_Sn have relatively good mechanical properties. It is superior to Sn in terms of melting temperature, Young’s modulus and hardness. In addition, Cu_3_Sn has a fracture toughness of 5.72 MPa/m, which is double the value of Cu_6_Sn_5_(2.80 MPa/m). Qiu et al.^10^ prepares single Cu_3_Sn solder joints using Cu plating with Sn films in one way by reflowing at 260 ℃ for 24 h (1 MPa auxiliary pressure) with a joint thickness of about 10 μm. In the other way, reflowing at 340 ℃ for 3 min (9.6 MPa auxiliary pressure) was used, but the joint thickness was only 3 μm. Others have worked similarly, using a sandwich structure (Cu/Sn/Cu) to obtain Cu_3_Sn solder joints by the TLP (transient liquid phase) method, which requires assisted application of pressure or ultrasound, or current^9,11–13^. Such solder joints are often only a few microns thick (less than 10 μm). For the thermal–mechanical reliability of the joint, a certain thickness (more than 15 μm) of the joint is desirable to alleviate the stress concentration^4–6^.

**Figure 1:**
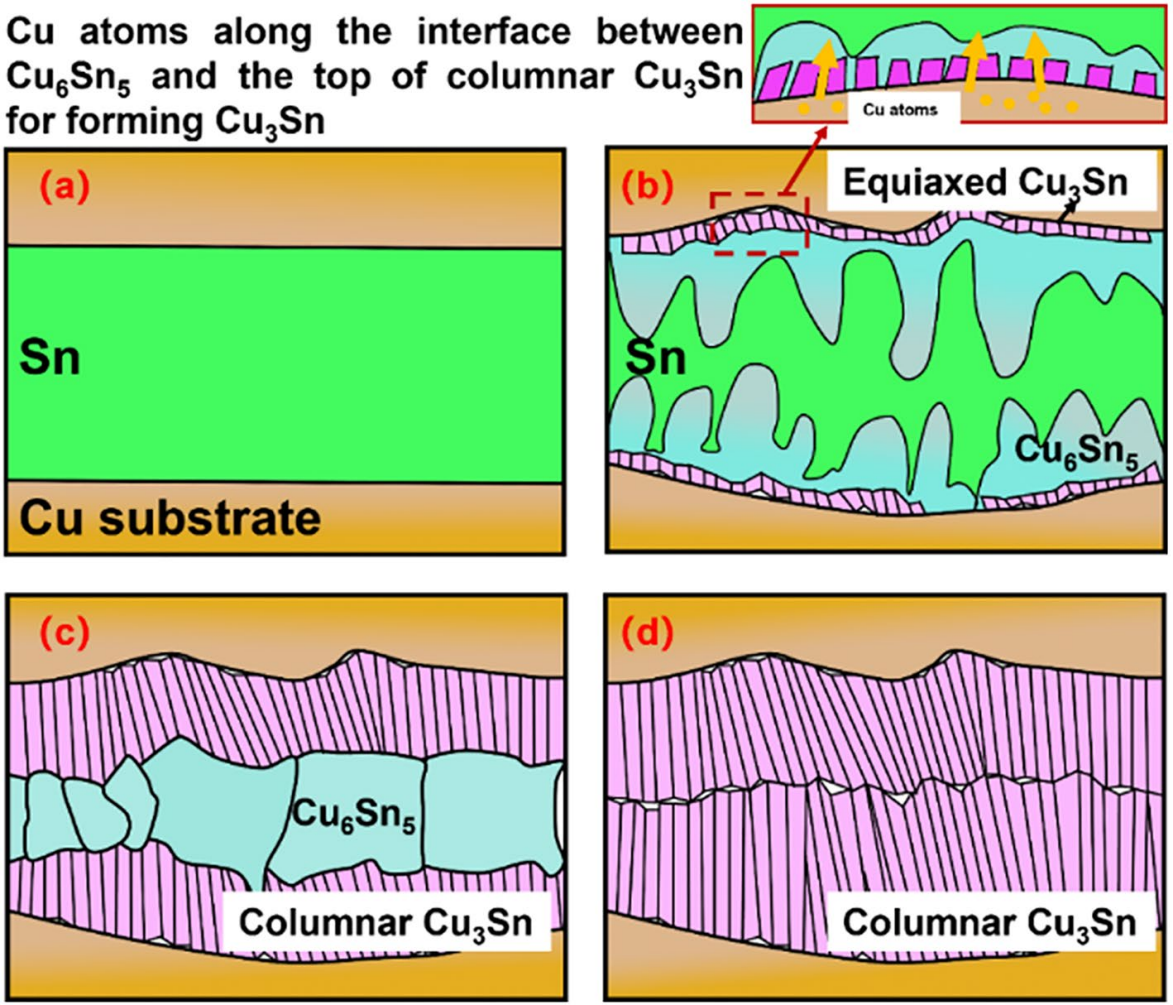
Schematic diagram of the structure of Cu_3_Sn solder joints prepared by the conventional TLP method (**a**) Cu/Sn/Cu sandwich structure, (**b**) Cu atoms along the interface between Cu_6_Sn_5_ and the top of columnar Cu_3_Sn for forming Cu_3_Sn, (**c**) Cu_3_Sn becomes longer, (**d**) Cu_3_Sn grains grow along the long axis of the columnar grains. When the opposite Cu_3_Sn grains touch each other, the grains stop growing, leaving a Cu_3_Sn boundary line in the middle of the joint.

Cu_3_Sn is an intermetallic compound with multiple morphologies. In recent years, there have been some studies on the different morphologies of Cu_3_Sn. Equiaxed Cu_3_Sn is the most studied grains at present. The Cu_3_Sn solder joints obtained by the conventional TLP (Cu/Sn/Cu sandwich structure) method are composed of coarse columnar Cu_3_Sn grains (Fig. 1). Past studies have concluded that during the soldering process, Cu_3_Sn grains are first nucleated in a fine isometric shape, this is because there is not enough time and space for complex shaped Cu_3_Sn grains to emerge. The Cu_3_Sn grains simply grow in an equiaxial shape, this is because the lowest energy is required to grow when the Cu_3_Sn grains are prevented from growing in their preferred growth direction. As the equiaxed Cu_3_Sn grains grow to a critical size, the Cu atoms along the Cu_3_Sn/Cu_6_Sn_5_ interface will participate in the interfacial reaction to form Cu_3_Sn, choosing to cross the parallel dense stacking planes of the Cu_6_Sn_5_ grains to obtain the least diffusion resistance. As a result, columnar Cu_3_Sn grains are formed, which means that the Cu_3_Sn grains change from an equiaxed shape to a columnar shape. However, caused by the different diffusion distances, Cu atoms diffuse into Cu_6_Sn_5_ forming Cu_3_Sn along the interface between Cu_6_Sn_5_ and the top of the columnar Cu_3_Sn. As a result, the columnar Cu_3_Sn grains continue to grow as the soldering proceeds, characterized by a greater increase in length than in width^7,8,12,14^.

In addition, Panchenko et al.^15^ in 2014 discovers a new morphology of Cu_3_Sn with porous-type. David et al.^16^ investigates the growth competition between layer-type (columnar Cu_3_Sn) and porous-type Cu_3_Sn in micro-bumps. The crystals of this Cu_3_Sn have been shown to form a superlattice with hexagonal symmetry (JCPDS Card No. 65-4653 ^16^.). The hexagonal plane is a low-energy plane. Since porous Cu_3_Sn has a very large free surface area, its lamellar surface has a low surface energy. It is possible that the lamellae form on the (002), (020), and (200) planes of Cu_3_Sn, and possibly on the superlattice plane. For this, a hypothesis was put forward. In the layer-type growth, they^16^ assume Cu to be the dominant diffusing species, coming from the Cu column. The Cu reacts with Cu_6_Sn_5_ to grow the Cu_3_Sn layer. In the porous-type growth, they^16^ assume Sn to be the dominant diffusing species, coming from the depletion of Sn in Cu_6_Sn_5_. The depleted Cu6Sn5 transforms to the porous-type Cu_3_Sn. At the same time, the Sn diffuses to the side-wall of Cu column to form a coating of Cu_3_Sn.The difference between the two morphologies of Cu_3_Sn comes from the diffusion of different atoms^17,18^. Morphology will affect the atomic diffusion during soldering, which will further influence the interfacial reaction during Soldering. In addition, morphology will affect the crack expansion path of the load-bearing joint and affect the reliability of the joint^12,19^.

In this manuscript, a Cu@Cu_6_Sn_5_ core–shell structured bimetallic Particle (approx. 1 μm in diameter) is successfully prepared and introduced as a new solder material for the packaging of power devices to obtain a Cu_3_Sn all-IMC solder joint. This solder joint is composed entirely of equiaxed Cu_3_Sn grains. With the help of Cu@Cu_6_Sn_5_ materials, the effect of different atomic diffusion (Cu atoms and Sn atoms) on the Cu_3_Sn morphology during the soldering process was investigated.

## Materials and methods

### Cu@Cu_6_Sn_5_ particles

To prepare the Cu@Cu_6_Sn_5_ core–shell particles, Cu particles (approx. 1 μm in diameter) with a particle size of 0.5–1.0 μm were used. A specific amount of cleaned Cu Particles and polyethylene glycol were dispersed completely in deionized water. Then, a reducing agent constituting sodium citrate, sodium hypophosphite, hydroquinone, and disodium EDTA in a mass ratio of 10:30:1:1 was added to the solution. Subsequently, a ligand CH_4_N_2_S was added to the solution. The amount of CH_4_N_2_S was adjusted such that the mass ratio of CH_4_N_2_S to Cu remained between 3:1 and 2:1. In another container, stannous chloride dihydrate was added to hydrochloric acid, followed by ultrasonication until the solution was clarified and transparent. The amount of stannous chloride was adopted such that the mass ratio of stannous chloride to Cu remained between 1:2 and 1:3. The stannous chloride solution was then added to the solution containing Cu Particles and stirred continuously for 50–90 min at room temperature to ensure a complete reaction. The reaction product was separated from the solution, repeatedly cleaned, and dried. The chemical reaction is as below:

$$\begin{aligned} & {\text{Sn}}^{ + } + {\text{Cu}} + 2({\text{CH}}_{4} {\text{N}}_{2} {\text{S}}) = {\text{Sn}} + [{\text{Cu}}({\text{CH}}_{4} {\text{N}}_{2} {\text{S}})_{2} ]^{2 + } \\ & 6{\text{Cu}} + 55{\text{n}} = {\text{Cu}}_{6} {\text{Sn}}_{5} \\ \end{aligned}$$

**Figure 2:**
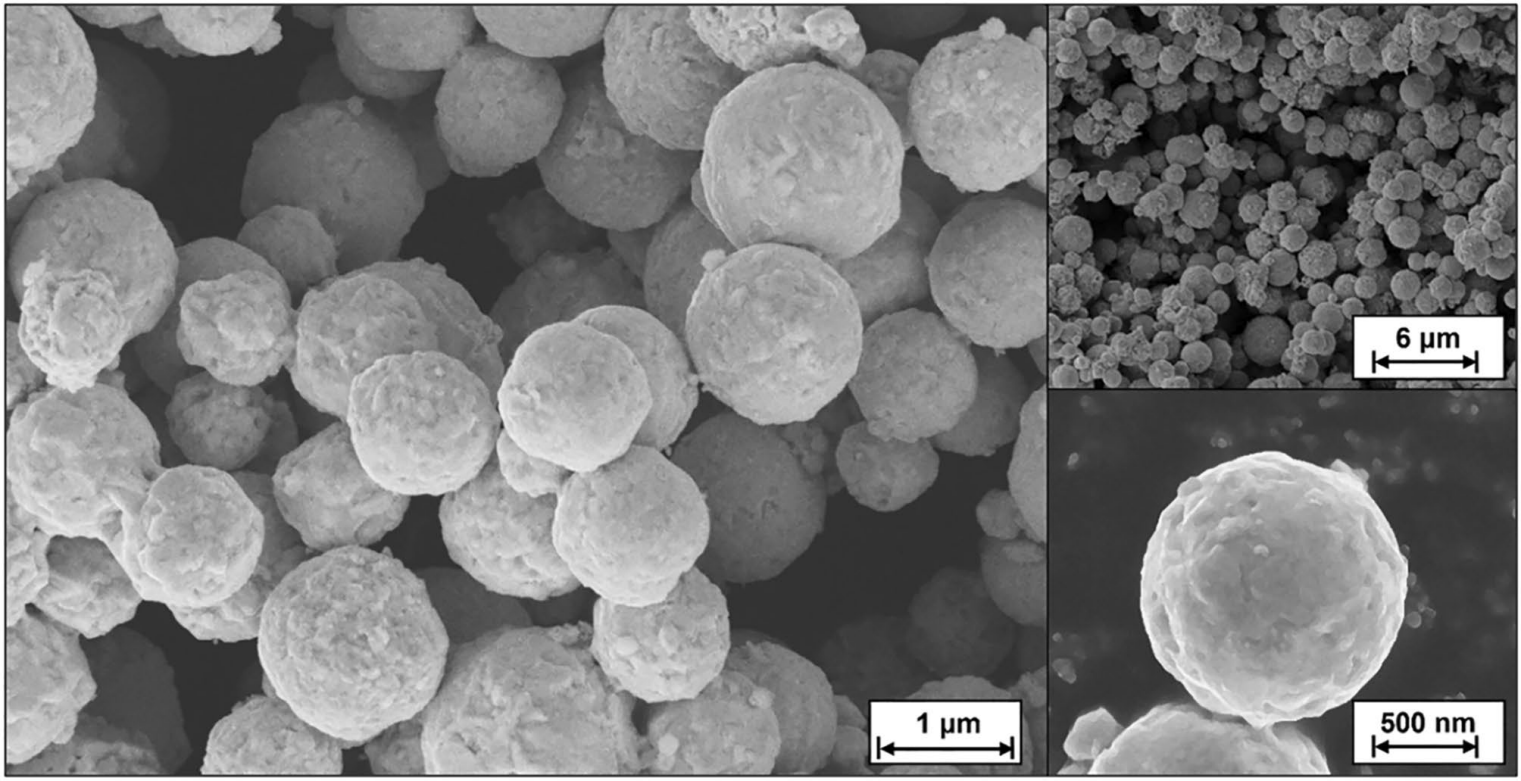
SEM images of Cu@Cu_6_Sn_5_ particles.

The heat given off by the reduction reaction accelerates this reaction (Fig. 2). The particles were characterized by XPS (Thermo, Scientific K-Alpha), SEM (FEI, FIB/SEM; HELIOS 600i), EDS (EDAX, XM4) and XRD (Rigaku, D/max 2800).

### Soldering

**Figure 3:**
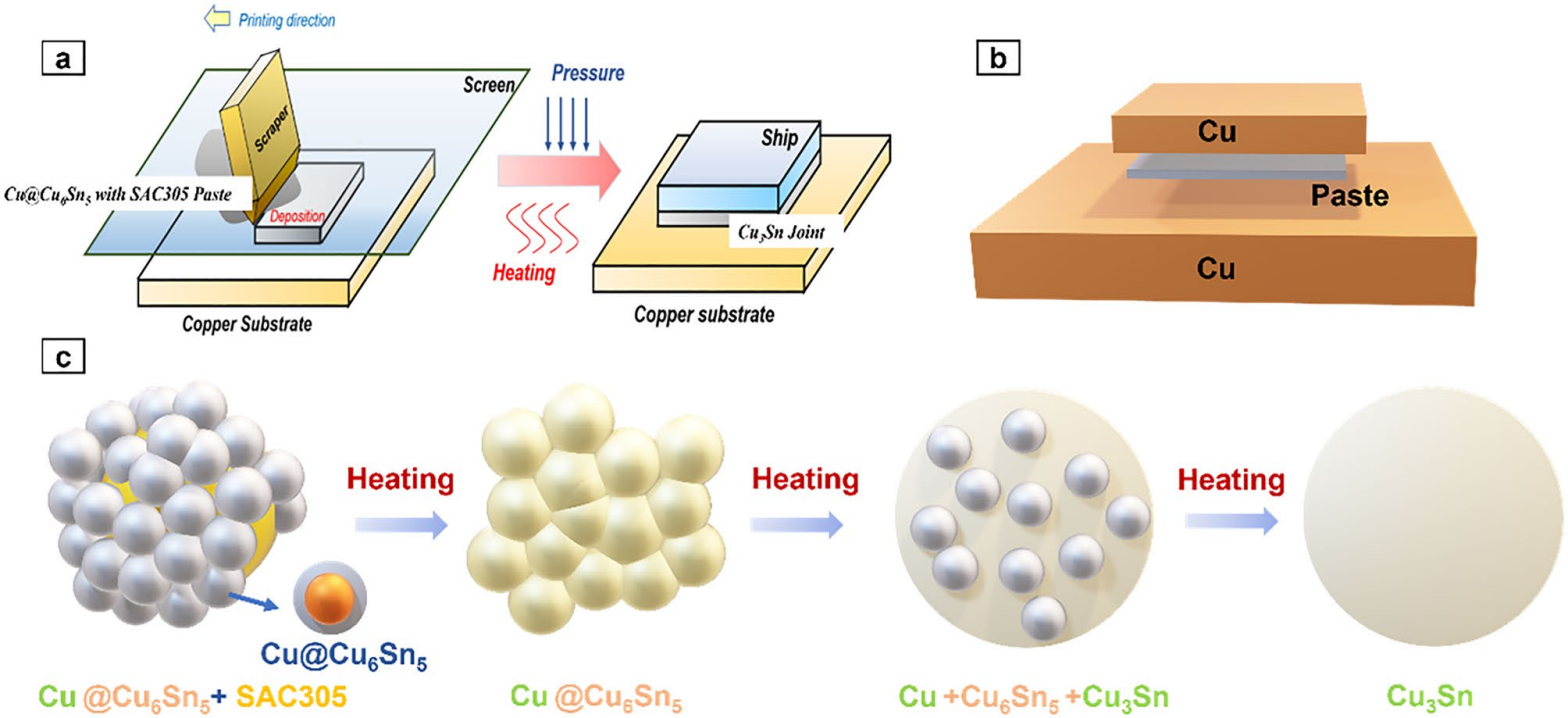
(**a**) The paste is screen-printed on the surface of a Cu substrate and reflowed at 280 °C under a pressure of 10 MPa for 60 min. (**b**) Transient liquid phase (TLP) bonding. (**c**) Schematic diagram of solder paste changes during heating. During the heating of the solder paste, Cu_6_Sn_5_ reacts with Cu to form Cu_3_Sn. In the early stage of reaction, the diffusion of Cu element dominates the reaction, and in the late stage, the diffusion of Sn atom dominates the reaction. This reaction process will cause the volume shrinkage of the joint, so the auxiliary pressure of 10 MPa shall be maintained during heating.

Ethyl cellulose and dibutyl phthalate were added to the pine oil alcohol solution and mixed under assisted sonication for 1 min. Then, a mixture of Span-85 and sulfosalicylic acid was added dropwise to the solution. The pine oil alcohol solution was mixed with Cu@Cu_6_Sn_5_ particle and SAC305 particles at a mass ratio of 2.8:1 to obtain a paste, which at this ratio, the atomic ratio of Cu to Sn in the paste is 3.2:1. The paste was screen-printed on the surface of a Cu substrate and reflowed at 280 °C under a pressure of 10 MPa for 60 min (Fig. 3a). It is worth noting that auxiliary pressure is necessary in the welding process because the process of Cu reacting with Cu_6_Sn_5_ to generate Cu_3_Sn is accompanied by volume shrinkage, which results in voids. Additional pressure is required to reduce the number of voids.

Based on the TLP bonding, the SAC305 melt filling reacts with the Cu-nuclei to generate Cu–Sn intermetallic compounds (IMCs) by heating and pressurizing. This reaction consumes the low-melting-point Sn phase and produces high-temperature solder joints. The bend surface of molten Sn is subjected to a certain additional pressure on the surface under the effect of surface tension.

The Cu–Sn interfacial chemical reaction is expressed as

$$\begin{aligned} & 6{\text{Cu}} + 5{\text{Sn}} \to {\text{Cu}}_{6} {\text{Sn}}_{5} \\ & {\text{Cu}}_{6} {\text{Sn}}_{5} + 9{\text{Cu}} \to 5{\text{Cu}}_{3} {\text{Sn}} \\ \end{aligned}$$

The rate of change in Gibbs free energy is the highest when the products adopt a scallop shape, which is favorable for the reaction. Therefore, Cu_6_Sn_5_ shows a scallop-type morphology. Considering the liquid solder during the soldering reaction as a binary solution system, where Cu is the solute and Sn is the solvent, the distribution of Cu in the liquid solder satisfies the Gibbs–Thomson effect. The difference in Cu concentration serves as the driving force for the diffusion of Cu in the soldering reaction, and the diffusion of Cu between adjacent IMC grains with different radii of curvature also leads to the incorporation of adjacent IMC grains. The microstructure undergoes a phase transformation in the order of Cu@η-Cu_6_Sn_5_ → ε-Cu_3_Sn. Eventually, the joint loses the typical characteristics of a core–shell structure and instead forms a uniform microstructure, as shown in Fig. 3.

The microstructure of solder joints and shear fracture surfaces were characterized using a focused ion beam/scanning electron microscope (FIB/SEM; HELIOS 600i; FEI) equipped with an electron dispersive X-ray detector (EDX; XM4; EDAX). The composition of shear fracture surfaces was characterized by X-ray diffractometry (XRD; D/max 2800; Rigaku). The melting points of the different phases in the solder joints were measured with a differential scanning calorimeter (DSC; STA 449F5; NETZSCH) at a heating rate of 10 °C s^−1^. The morphology of joint/Cu interface was observed by transmission electron microscopy (TEM, TecnaiG2F30, FEI).

And the grain orientation and grain sizes distribution of Cu_3_Sn was analyzed by Electron Backscattered Diffraction (EBSD, Nordly max3, Oxford).

To verify the long-term service reliability of the solder joints at high temperatures, the samples were subjected to aging tests at 300 °C using a muffle furnace, and the joint and mechanical properties of the samples were examined at 300, 600, 900, and 1200 h, respectively. A creep tester (SANS, GWTA-105, 100 kg) was used to measure the shear strength of the welded joints at room temperature at a shear rate of 0.25 mm s^−1^. The sheared sample is a 5 × 5 × 2 (mm) copper substrate soldered to a 10 × 10 × 2 (mm) copper substrate (Fig. 3).

## Results and analysis

### ***Cu@Cu***_***6***_***Sn***_***5***_*** particles***

**Figure 4:**
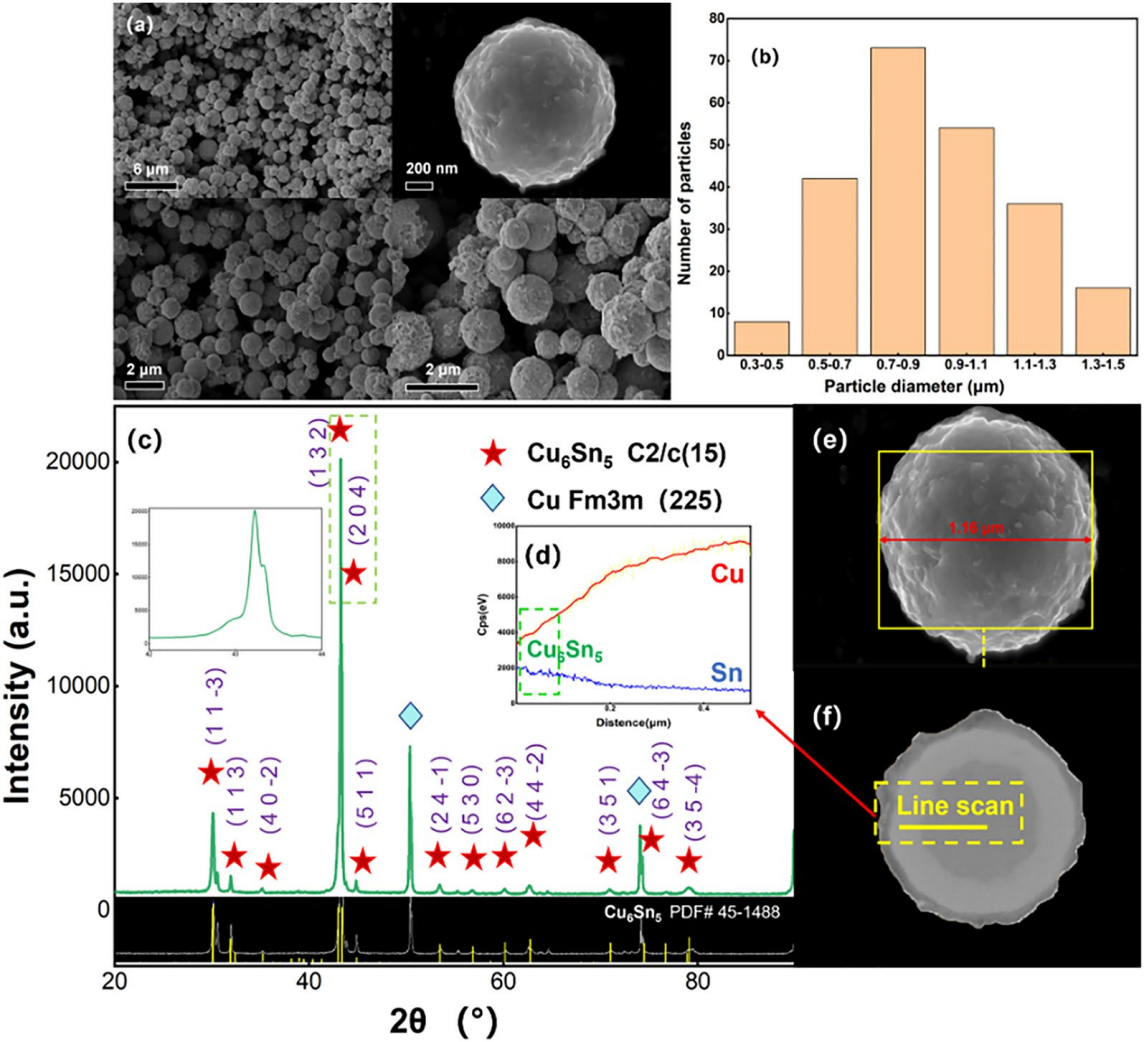
SEM images of Cu@Cu_6_Sn_5_ (**a**, **e**) SEM image of Cu@Cu_6_Sn_5_ particles, (**b**) diameter length distribution statistics of particles, (**c**) XRD spectra of Cu@Cu_6_Sn_5_ particles, (**d**) EDX result of Cu@Cu_6_Sn_5_, which is obtained from the line scan illustrated in (**f**).

Statistically, the diameter length of the particles is mainly distributed between 0.5 and 1.3 μm (Fig. 4a,b). The XRD pattern results of the particles showed that the surface of the particles is η-Cu_6_Sn_5_, and the EDX results also support this conclusion (Fig. 4c). SEM images show that Cu_6_Sn_5_ on the surface exhibits a scallop-like character (Fig. 4e). After chemical plating, the scallop-shaped shell covers the surface of the smooth Cu core. As shown in Fig. 4f, an EDX scan analysis is performed at the Cu–Cu_6_Sn_5_ interface, the average diameter of the Cu particles was 600 nm, and the thickness of the shell is about 200 nm (radial difference), Cu atoms diffuse throughout the shell (Fig. 4d). Because smaller copper particles have higher surface activity energy, the chemical reaction between the Cu core and the Sn layer is accompanied in the process of electroless Sn plating to generate Cu_6_Sn_5_.

### Evolution of the joint in soldering

**Figure 5:**
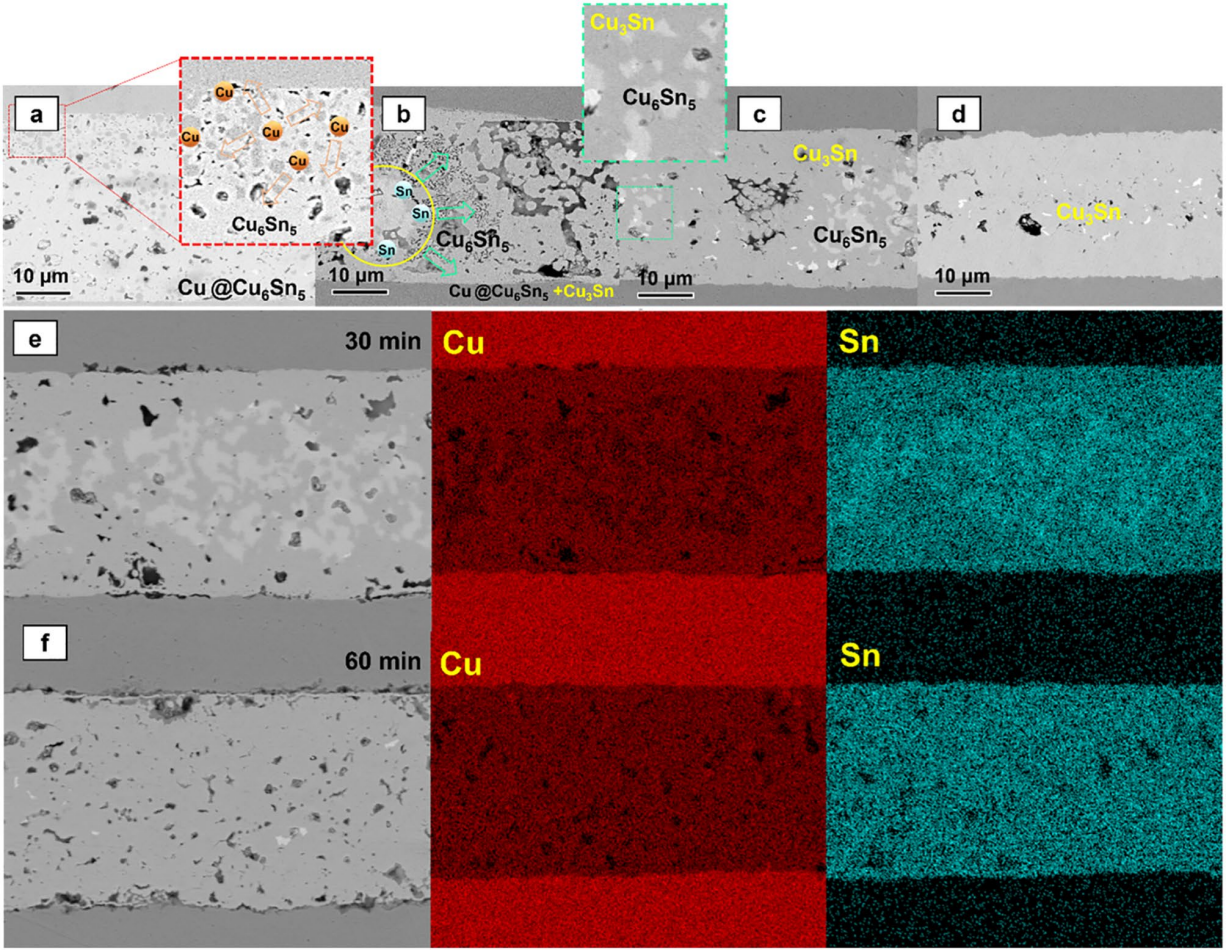
The change in joint tissue during reflow at 280 °C (10 MPa) (**a**) 5 min, (**b**) 15 min, (**c**) 30 min, (**d**) 60 min, (**e**) EDX mapping result of joint reflowing 30 min at 280 °C, (**f**) EDX mapping result of joint reflowing 60 min at 280 °C.

The microstructure undergoes a phase transformation in the order of Cu@Cu_6_Sn_5_ + SAC305 → Cu@Cu_6_Sn_5_ + Cu_3_Sn → Cu_3_Sn. Eventually, the joint loses the typical characteristics of a core–shell structure and instead forms a uniform microstructure. The joint reflowing 30 min and 60 min are analyzed using Scanning SEM coupled with energy dispersive X‐ray spectroscopy (EDX) to confirm the transformation of the binary system (Fig. 5). The EDX results demonstrate the process of second stage diffusion.

**Figure 6:**
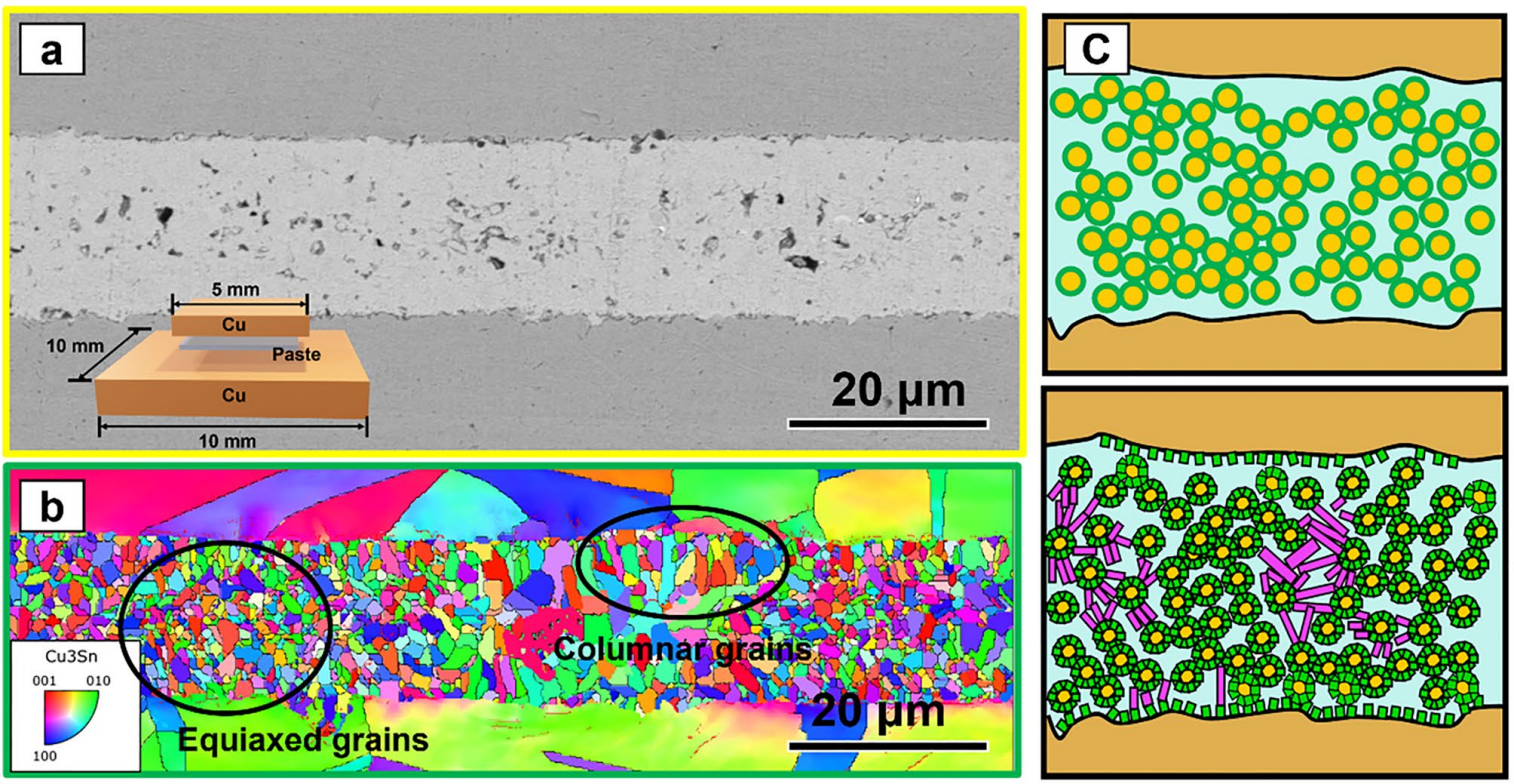
EBSD mapping of the joint (10 MPa auxiliary pressure, 60 min reflow at 280 ℃) (**a**) SEM image of joint reflowed 60 min at 280 ℃, (**b**) EBSD mapping, (**c**) schematic diagram of the growth of Cu_3_Sn.

During the reflow of the Cu@Cu_6_Sn_5_ core–shell particles with SAC305, the reaction takes place in two stages. First stage, the SAC305 reacts with Cu to form Cu_6_Sn_5_ IMCs. In this stage, Cn atoms diffuse from the core–shell particles through the Sn melt throughout the joint and react with the Sn melt to form Cu_6_Sn_5_. The Cu_6_Sn_5_ nucleation event occurs at the solid–liquid phase interface, i.e., the Cu_6_Sn_5_/Sn interface. The growth of Cu_6_Sn_5_ at this stage is dominated by grain boundary diffusion. The grain boundary diffusion is very fast, so the Sn melt disappears very quickly. In experiments, that auxiliary pressure and high temperatures accelerate the process (280 ℃, 10 Mpa), it only takes about 5 min (Fig. 6a) and there is almost no residual Sn melt in the joint.

In the second stage, The Sn atoms also diffuse, and the Cu atoms continue to diffuse. The remaining Cu atoms diffuse and react with Cu_6_Sn_5_ to form Cu_3_Sn. In this joint, the Cu_3_Sn nucleation events are more complex compared to the conventional sandwich TLP method (Figs. 1, 8a,b). Nucleation of Cu_3_Sn occur over two interfaces, the Cu/Cu_6_Sn_5_ interface, and the Cu_6_Sn_5_/Cu_3_Sn interface, respectively. The nucleation at different interfaces results in different Cu_3_Sn grain morphologies. The number of equiaxed grains is much higher than that of columnar grains in this joint obtained by having Cu@Cu_6_Sn_5_ reflow. Thus, the number of columnar crystals is positively correlated with the percentage of SAC305 in the solder paste.

**Figure 7:**
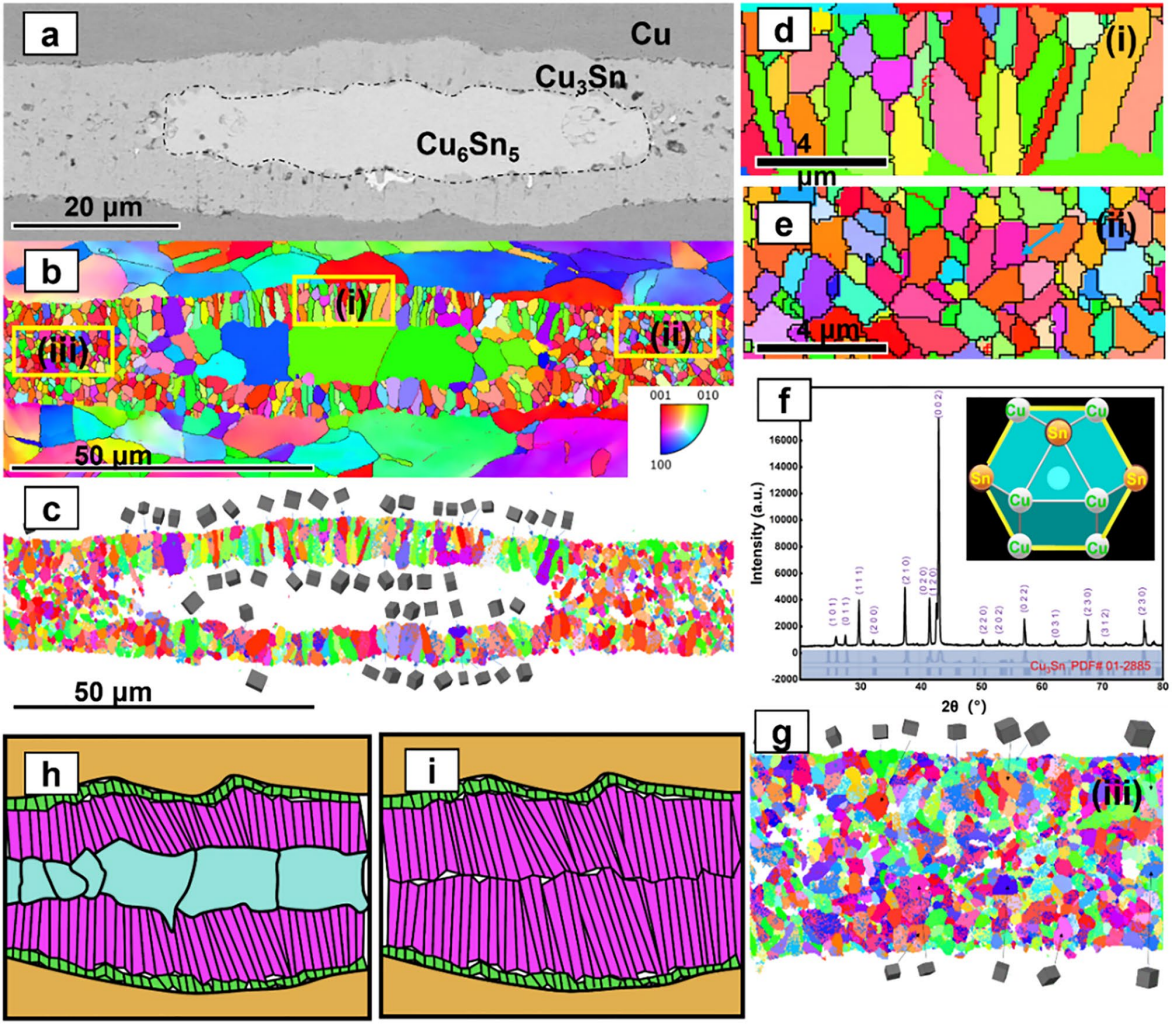
EBSD mapping of the joint (10 MPa auxiliary pressure, 30 min reflow at 280 ℃) (**a**) SEM image of joint reflowed 30 min at 280 ℃, (**b**) EBSD mapping, (**c**) EBSD mapping, (**d**) EBSD mapping of columnar grains (location(i) in Fig. 7-b ), (**e**) EBSD mapping of equiaxed grains (location(ii) in Fig. 7-b ), (**f**) inverse pole figure. (**g**) EBSD mapping of location(iii) in Fig. 7-b.

**Figure 8:**
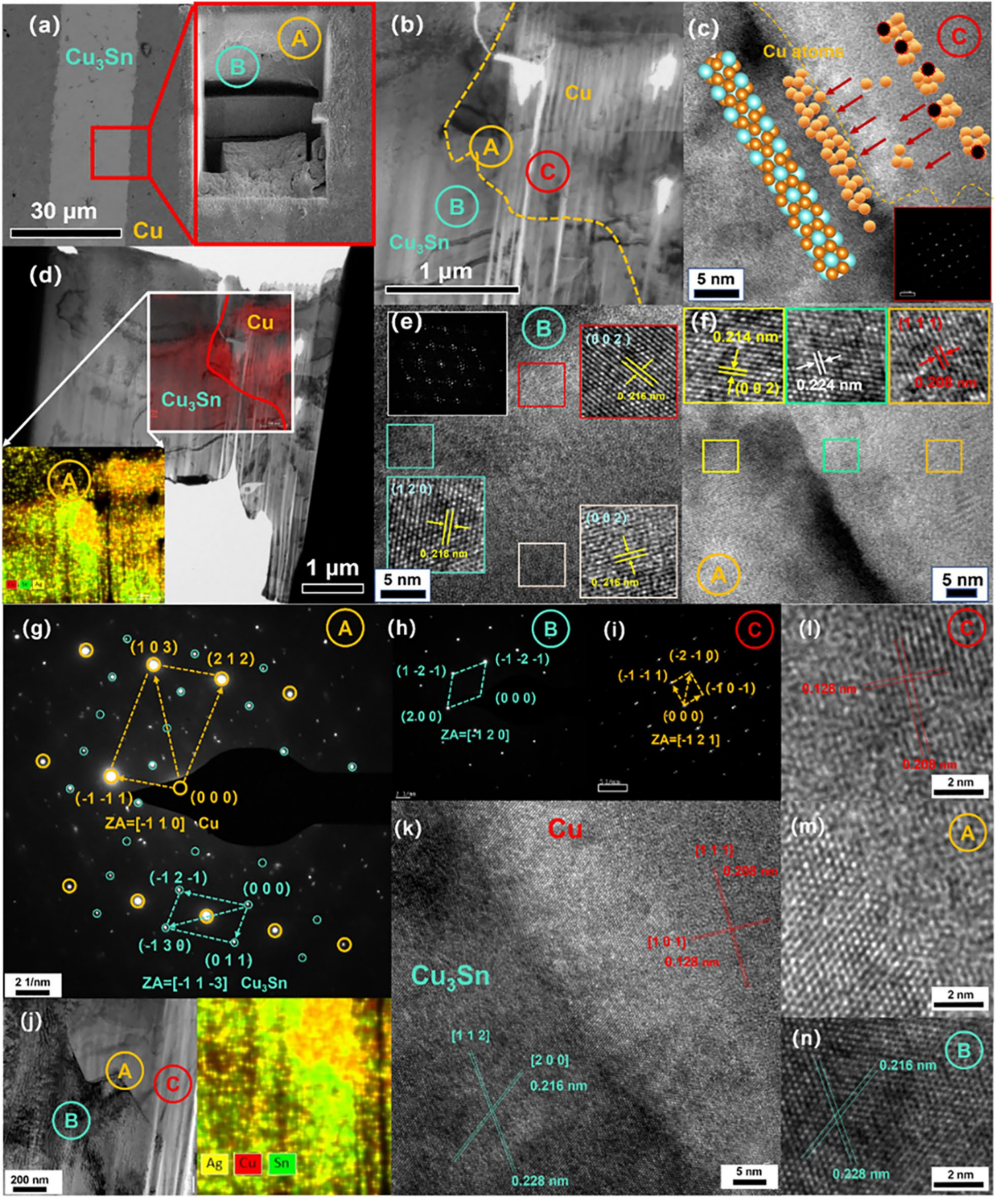
(**a**) TEM (transmission electron microscope) images of solder joint after reflowing at 280 °C for 60 min, (**b**) TEM images at the Cu/Cu_3_Sn interface. (**c**) The high-resolution images at the Cu/Cu_3_Sn interface. (**d**) TEM images at the Cu/Cu_3_Sn interface. (**e**) The high-resolution images of Cu_3_Sn. (**f**) The high-resolution images at the Cu/Cu_3_Sn interface. (**g**) The electron diffraction pattern of the interface. (**h**) The electron diffraction pattern of the Cu_3_Sn. (**i**) The high-resolution image pattern of the Cu. (**j**) The electron diffraction pattern of the Cu_3_Sn in the interface. (**k**) The high-resolution images at the Cu/Cu_3_Sn interface. (**l**) The high-resolution images at the Cu/Cu_3_Sn interface. (**m**) The high-resolution images at the Cu/Cu_3_Sn interface. (**n**) The high-resolution images at the Cu/Cu_3_Sn interface.

**Figure 9:**
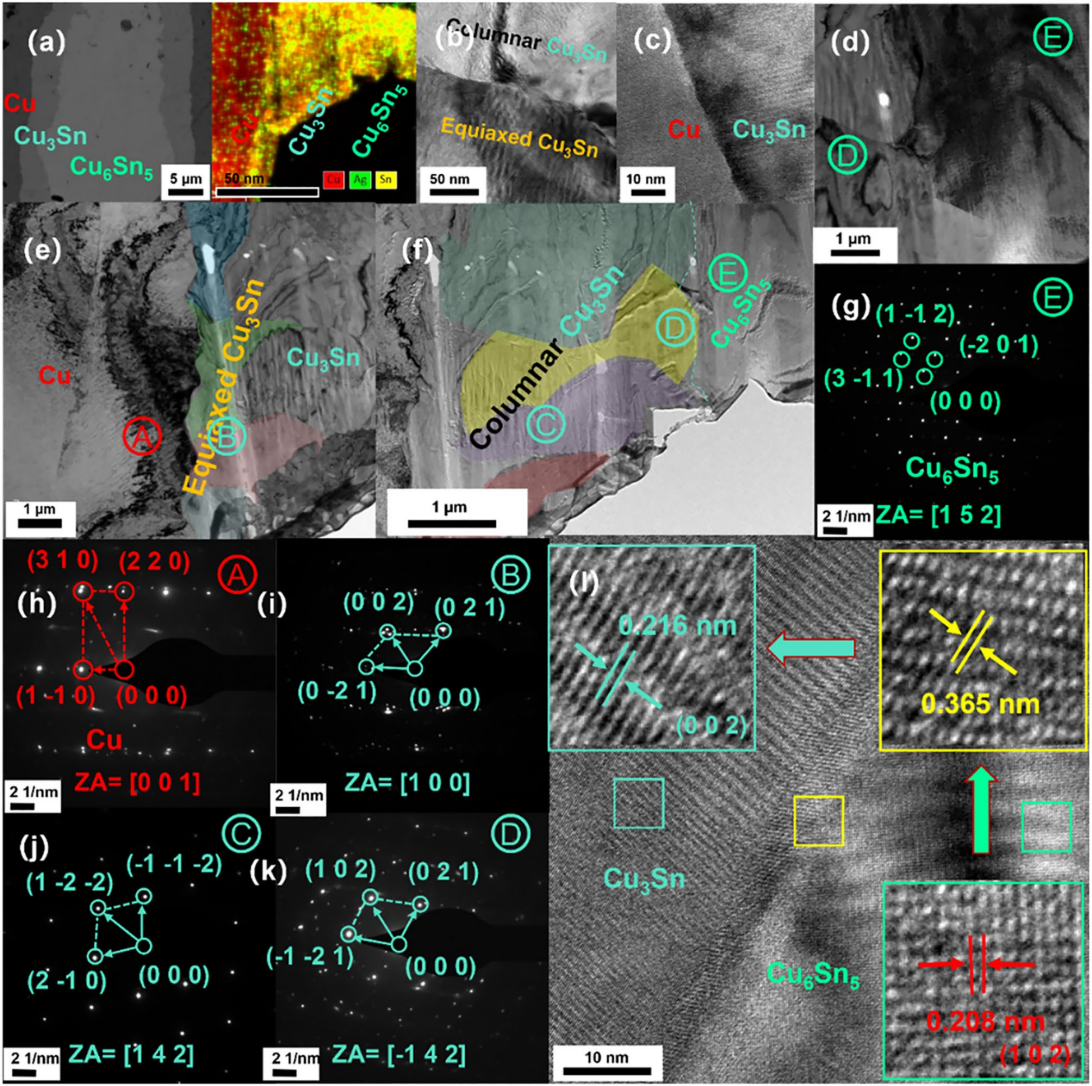
TEM (transmission electron microscope) images of solder joint after reflowing at 280 °C for 30 min (**a**) SEM images at the Cu/Cu_3_Sn/Cu_6_Sn_5_ interface. (**b**) TEM images at the Cu_3_Sn/Cu_3_Sn interface. (**c**)The high-resolution images at the Cu/Cu_3_Sn interface. (**d**) TEM images at the Cu_3_Sn/Cu_6_Sn_5_ interface. (**e**) TEM images of equiaxed Cu_3_Sn. (**f**) TEM images of columnar Cu_3_Sn. (**g**) The electron diffraction pattern of the Cu_6_Sn_5_. (**h**) The electron diffraction pattern of the Cu (**i**) The electron diffraction pattern of the Cu_3_Sn. (**j**) The electron diffraction pattern of the Cu_3_Sn. (**k**) The electron diffraction pattern of the Cu_3_Sn. (**l**) The high-resolution images at the Cu_6_Sn_5_/Cu_3_Sn interface.

Two different morphologies, one with equiaxed grains (Figs. 6, 7) and one with columnar grains (Fig. 6), are obtained by observing electron backscattering diffraction (EBSD) mapping with different nucleation interfaces. In Fig. 7b, the Cu_3_Sn grains on the top side near the Cu/Cu_3_Sn interface are equiaxed grains, while those on the bottom side near the Cu_3_Sn/Cu_6_Sn_5_ interface are columnar grains. The TEM mapping and electron diffraction pattern results (Figs. 8, 9) show that the two morphologies of Cu_3_Sn have the same crystal structure. The grains of both morphologies have the same crystal structure—the space group of the crystal is cmcm(63).

### Two different morphologies of Cu_3_Sn

Two different morphologies of Cu_3_Sn are observed in the experiment, equiaxed and columnar (Fig. 6). The Cu_3_Sn phase arises from a solid-state reaction between Cu and Cu_6_Sn_5_, which is diffusion controlled. The reaction of the Cu-Sn binary system is controlled by the Gibbs free energy change rate, and the reaction path of the system tends to have the largest Gibbs free energy change rate $(\Delta G)$^20,21^.

$$\Delta G = \int_{0}^{\tau } {(dG{/}dt)dt = - \int_{0}^{\tau } {Fv\,dt} }$$

*F*: reaction driver; *v*: reaction rate; τ: reaction time.

A further study by Paul^22^ updated the ratio of Cu@Cu_6_Sn_5_ interdiffusion coefficients and found that in Cu_3_Sn, Cu is the dominant diffusing particle, while in Cu_6_Sn_5_, the diffusion of Sn is slightly faster than Cu in Cu_6_Sn_5._

The growth of equiaxed Cu_3_Sn grains is a ripening process, which is dominated by the diffusion of copper atoms from the copper substrate to the Cu_6_Sn5/Cu_3_Sn interface to form Cu_3_Sn^7,8,12,14^. Refs.^23,24^ after analyzing their systematic experimental data on retarded Cu_3_Sn formation, concluded that "nucleation rather than growth is the cause of Cu_3_Sn deficiency." This new insight distinguishes from all previous studies on Cu_3_Sn fabrication, which focused on stimulating Cu_3_Sn growth rather than nucleation. Thus, it provides a new fundamental clue to the fabrication of Cu_3_Sn. Simulation of several Cu3Sn superstructures reveals that the presence of anti-phase boundaries can change the transport anisotropy by ~ 10%. The DFT thermodynamic stability analysis suggests that the previously observed D019 structure featuring the maximum number of anti-phase boundaries is the Cu_3_Sn ground state in the relevant temperature range, which points to the importance of kinetic factors in the formation of the known long-period superstructures^7,22,24^.

The Cu_3_Sn grains appearing along the Cu/Cu_6_Sn_5_ interface were found to have different grain orientations by electron backscatter diffraction techniques (Fig. 7). On this basis, these equiaxed Cu_3_Sn grains formed after nucleation also have different grain orientations. The orientation of a grain depends on the arrangement of the atoms within that grain. This means that the atomic arrangements between equiaxed Cu_3_Sn grains are different. Due to the difference in atomic arrangement, equiaxial Cu_3_Sn grains need to grow in different directions to obtain the lowest energy. However, for each equiaxed Cu_3_Sn grain, its growth along the preferred growth direction is hindered by its neighboring Cu_3_Sn grains. Although the growth of Cu_3_Sn grains along their preferred growth direction is prevented, the growth of Cu_3_Sn grains does not stop. This means that the Cu_3_Sn grains must grow in other ways. Initially, the Cu_3_Sn grains seek to grow in other directions. Of course, more energy is required to grow along these non-preferred directions. However, the possibility exists that the energy required to nucleate a new Cu_3_Sn grain shape may be lower compared to the energy required to grow along these non-preferred directions as well as to grow in other ways^14,25^.

The diffusion rate of Cu atoms in Cu_6_Sn_5_ is much smaller than the diffusion rate of Cu atoms in Sn. Therefore, when Cu_3_Sn is nucleated at the Sn/Cu_6_Sn_5_ interface, the growth of Cu_3_Sn is dominated by the diffusion of Sn atoms, and Cu_3_Sn grows in the direction of the lowest energy. In contrast, when Cu_3_Sn is nucleated at the Cu/Cu_6_Sn_5_ interface, the Sn atom diffusion dominates the growth of Cu_3_Sn, which is more likely to grow along all isometric directions and is easily nucleated. The SAC305 melt provided fast diffusion channels for Sn atoms as well as Cu atoms during the pre-reaction stage. The Cu@Cu_6_Sn_5_ particles provided many Cu/Cu_6_Sn_5_ interfaces, allowing equiaxed grains to form rapidly and making it difficult to grow. In a small number of regions in the joint, the enrichment Cu_6_Sn_5_ results in the formation of columnar grains at the Cu_6_Sn_5_/Cu_3_Sn interface. That is, Cu_3_Sn tends to nucleate more at the Cu/Cu_6_Sn_5_ interface, while Cu_3_Sn nucleated at the Cu_6_Sn_5_/Cu_3_Sn interface tends to grow into columnar grains.

In columnar-type growth, Sn is the dominant diffusing species, which comes from the depletion of Sn in Cu_6_Sn_5_. The depleted Cu_6_Sn_5_ is transformed into columnar Cu_3_Sn. In equiaxed-type growth, Cu is the dominant diffusing species. Cu reacts with Cu_6_Sn_5_ to grow a Cu_3_Sn layer. In this process, the equiaxed grains grow in preference to the columnar grains. Different diffusion modes of different atoms affect the lattice type and change the morphology of Cu_3_Sn. We confirmed this conclusion by observing the orientation relationship.

### Orientation relationship

TEM mapping is performed on the joints obtained after reflowing of two solder paste, respectively. One is solder joint after reflowing at 280 °C for 60 min (Cu/Cu_3_Sn interface Fig. 8), and the other is solder joint after reflowing at 280 °C for 30 min (Cu/Cu_6_Sn_5_/Cu_3_Sn interface Fig. 9).

Cu_3_Sn was reported as an ɛ-phase with a Cu_3_Ti-type^26^. The electron diffraction pattern is taken from the Cu_3_Sn equiaxed grains phase in different directions (Fig. 8). In the diffraction pattern, the stronger spots correspond to the main reflections of the basic hexagonal lattice, while the weaker additional spots, appearing at half the distance between the main reflections, correspond to the superlattice reflections of the superstructure of the basic hexagonal lattice. The equiaxed Cu_3_Sn grains have an orientation relationship with the Cu substrate, and the orientation of the Cu substrate affects the Cu_3_Sn grains nucleated at the Cu/Cu_6_Sn_5_ interface. The electron diffraction pattern shows site-orientation relationships: Cu [**− 1 1** 0]//Cu_3_Sn [− **1 1 − 3**] (Fig. 8g), Cu [**− 1 2** 1]//Cu_3_Sn [**− 1 2 0]** (Fig. 8h,i), Cu [**1 1** 1]//Cu_3_Sn [**1 1 2]** (Fig. 8k). Caused by the poor interdiffusion coefficients of Cu atoms in Cu_3_Sn, a large number of Cu atoms gather and accumulate at the Cu_3_Sn/Cu interface (Fig. 11c,f), and the lattice structure on the copper side has also been damaged (Fig. 8m). This confirms the nucleation of equiaxed Cu_3_Sn grains at the Cu/Cu_6_Sn_5_ interface, dominated by the diffusion of Cu atoms.

There is no orientation relationship observed either between equiaxed Cu_3_Sn grains and columnar Cu_3_Sn grains (Fig. 9i,j). This suggests that the columnar Cu_3_Sn grains is developed thermally during the solidification of the Cu–Sn alloy. However, the columnar Cu_3_Sn grains have a orientation relationship with the Cu_6_Sn_5_ grains: Cu_6_Sn_5_ [1 0 2]//Cu_3_Sn [0 0 2],:Cu_6_Sn_5_ [1 5 2]//Cu_3_Sn [1 4 2]. The above orientation relationship once again confirms our proposed hypothesis that in columnar-type growth, Sn is the dominant diffusing species, which comes from the depletion of Sn in Cu_6_Sn_5_. The depleted Cu_6_Sn_5_ is transformed into columnar Cu_3_Sn. The antiphase boundaries (APB) structure was observed in the columnar Cu_3_Sn region (Fig. 9i,k). The antiphase boundaries can be described as larger orthorhombic unit cells with extended dimensions in the b-axis. APBs in columnar Cu_3_Sn crystals are observed. This is because the APB superstructure is based on the Cu_3_Ti-type lattice, which is orthorhombic.

### Shear strength and fracture

**Figure 10:**
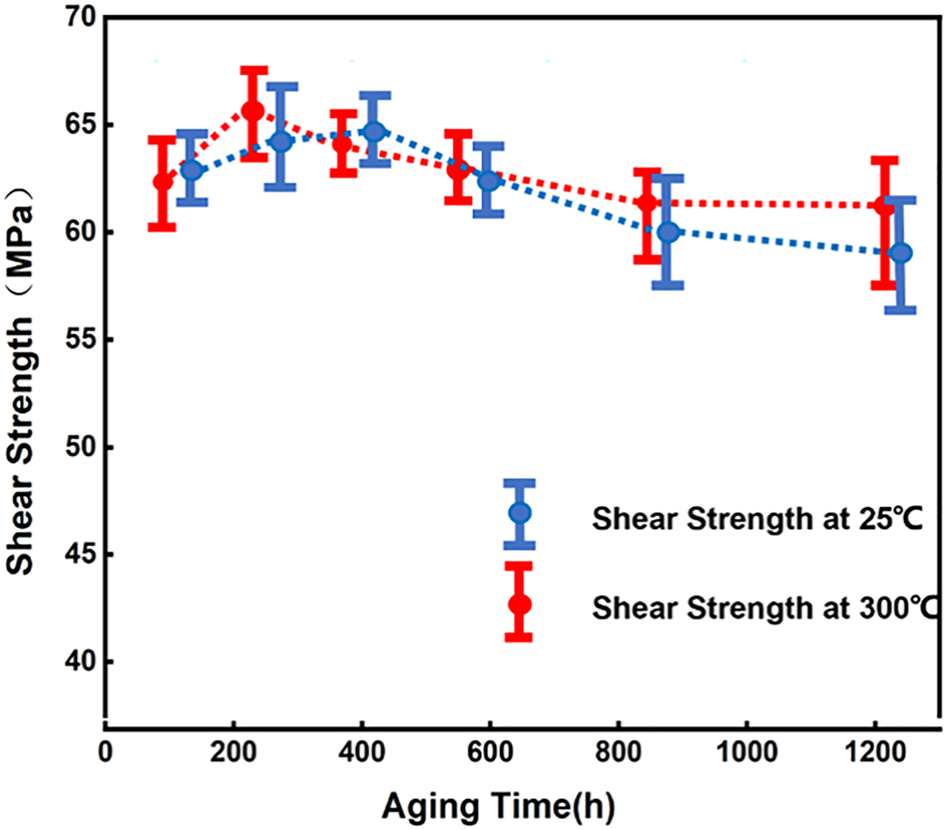
Shear strength of Cu_3_Sn joint as a function of aging time.

Shear experiments (Fig. 10) revealed that the shear strength of the joint is approximately 63.2 MPa and 65.2 MPa at room temperature and 300 °C, respectively. The strength of this joint is stronger than those made with most of the current soldering joint materials (SAC-305, Sn–Bi, etc.) and much higher than their service temperatures.

**Figure 11:**
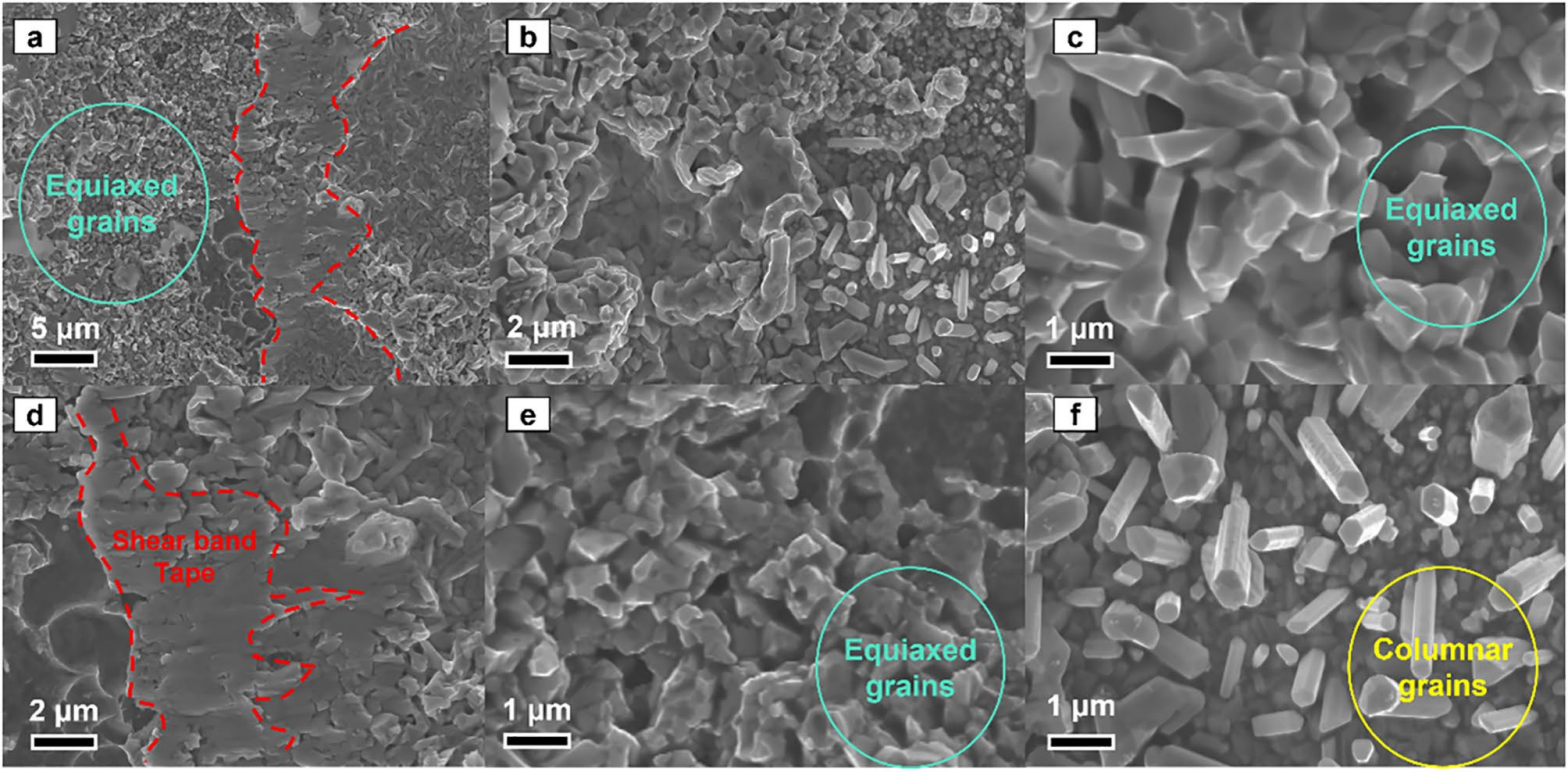
SEM image of fracture morphology (**a**) shear bands exist in the mixed zone of equiaxed grains and columnar grains, (**b**) equiaxed grains, (**c**) equiaxed grains, (**d**) shear band tape, (**e**) equiaxed grains, (**f**) columnar grains.

Notably, the formation of Cu_3_Sn is often accompanied by volume shrinkage, and hence, the Cu_3_Sn phase often contains numerous cavities. An auxiliary pressure of 10 MPa is applied to the joint during the soldering process, which significantly reduced the number of voids in the joint. In the aging experiments at 300 °C, the organization and properties of the joints remained unchanged even after 600 h. The shear fracture of the unaged sample is analyzed. The fracture cross section is mainly composed of equiaxed Cu_3_Sn grains (Fig. 11a,c,e), and the fracture mode is plastic intergranular fracture, and shear band tape exists on the fracture surface (Fig. 11b,f). Columnar Cu_3_Sn grains are also found in the fracture, distributed only in a very small area. Under shear stress, the plastic deformation of Cu_3_Sn grains is highly localized, forming micron-scale shear bands; the formation and rapid expansion of shear bands induce macroscopic brittle fracture of the joint (Fig. 11d).

## Conclusions

Cu@Cu_6_Sn_5_ core–shell particles (1 μm) are prepared by the chemical reduction method.

A solder paste is obtained by mixing Cu@Cu_6_Sn_5_ particles with SAC305 in a 2.8:1 mass ratio and adding pine oil alcohol. This solder paste is reflowed at 280 °C and 10 MPa auxiliary pressure for 60 min to obtain a joint composed entirely of Cu_3_Sn. The joint consisted mainly of equiaxed Cu_3_Sn grains, and a small portion of columnar Cu_3_Sn grains. The reason why the joints are mainly composed of equiaxed Cu_3_Sn grains is because the Cu@Cu_6_Sn_5_ particles provide enough Cu/Cu_6_Sn_5_ interface.

In columnar-type growth, Sn is the dominant diffusing species, which comes from the depletion of Sn in Cu_6_Sn_5_. The depleted Cu_6_Sn_5_ is transformed into columnar Cu_3_Sn. In equiaxed -type growth, Cu is the dominant diffusing species. Cu reacts with Cu_6_Sn_5_ to grow a Cu_3_Sn layer.

The equiaxed Cu_3_Sn grain nucleates at the Cu/Cu_3_Sn interface have an Orientation Relationship with the Cu substrate. Columnar Cu_3_Sn grains at the Cu_6_Sn_5_/Cu_3_Sn interface have an Orientation Relationship with Cu_6_Sn_5_. This confirms the previous conclusion.

After reflowing, the solder joints transformers to a single-phase Cu_3_Sn IMC. Shear experiments on the joints reveal recorded a shear strength of approximately 63.2 MPa and 65.2 MPa at room temperature and 300 °C, respectively.

## Data Availability

The datasets used and/or analysed during the current study available from the corresponding author on reasonable request.
